# Risk factors and nomogram for the prediction of intracranial hemorrhage in very preterm infants

**DOI:** 10.1186/s12887-024-05274-0

**Published:** 2024-12-04

**Authors:** Yan Wang, Yong Yang, Lijun Wen, Minxu Li

**Affiliations:** Department of Neonatology, Dongguan Maternal and Child Health Care Hospital, Dongguan, 523000 China

**Keywords:** Very preterm neonates, Intracranial hemorrhage, Risk factors, Nomogram, Prediction model

## Abstract

**Aims:**

This study aims to identify important risk factors for intracranial hemorrhage (ICH) in very preterm infants at our institution and develop a predictive nomogram for early detection of ICH.

**Methods:**

We retrospectively analyzed neonates with a gestational age (GA) under 32 weeks, admitted to the neonatal intensive care unit from March 2022 to July 2023. Infants were categorized into two groups based on ultrasound findings and assessed for thirteen variables including gender, GA, birth weight (BW), acidosis, among others. We used multivariate logistic regression analysis to build a prediction model and identify independent risk factors for ICH. We build a prediction model by assigning 241 cases to the training set and 103 to the validation set (ratio 7:3).

**Results:**

Among 344 very preterm infants, the incidence of ICH was 36.9% (89 cases) in training set. Significant differences were observed in gestational age, birth weight, antenatal corticosteroids, mechanical ventilation days, and acidosis between cases and controls. Logistic regression analysis identified gestational age (OR = 0.674), antenatal corticosteroids (OR = 0.257), acidosis (OR = 2.556), and mechanical ventilation mechanical ventilation days(OR = 0.257) as independent risk factors for ICH. The C-index of the training and validation sets was 0.814 (95% CI: 0.762–0.869) and 0.784 (95% CI: 0.693–0.875), respectively. According to decision curve analysis, our model outperformed the “None” and “All” baseline lines over a wide range of risk thresholds (0.12–0.92).

**Conclusion:**

Acidosis and mechanical ventilation are independent risk factors for ICH in very preterm neonates, while higher gestational age and antenatal corticosteroid use are protective. The nomogram developed from these four factors demonstrates strong predictive accuracy and calibration, which can aid clinicians in identifying preterm infants at high risk for ICH and facilitate early diagnosis and management.

**Supplementary Information:**

The online version contains supplementary material available at 10.1186/s12887-024-05274-0.

## Introduction

Neonatal intracranial hemorrhage (ICH) is the most common form of brain injury in neonates. Severe cases of ICH have a mortality rate of 3.70%~44.68% [1]. Survivors often suffer from neurological sequelae, such as cerebral palsy, epilepsy, motor disorders, and cognitive impairments. With advancements in neonatal intensive care technologies, an increasing number of infants with low gestational ages are surviving, which, in turn, escalates the risk of brain hemorrhages and subsequent complications. Currently, research into the risk factors for intracranial hemorrhage in preterm infants varies across institutions, leading to discrepancies in reported outcomes. Our study focuses on very preterm infants, defined as those with a gestational age range of 28 weeks to 32 weeks, managed at our facility to build a prediction model. This comprehensive analysis will contribute to the prevention of ICH in preterm infants.

## Data and methods

### Study population

**Fig. 1:**
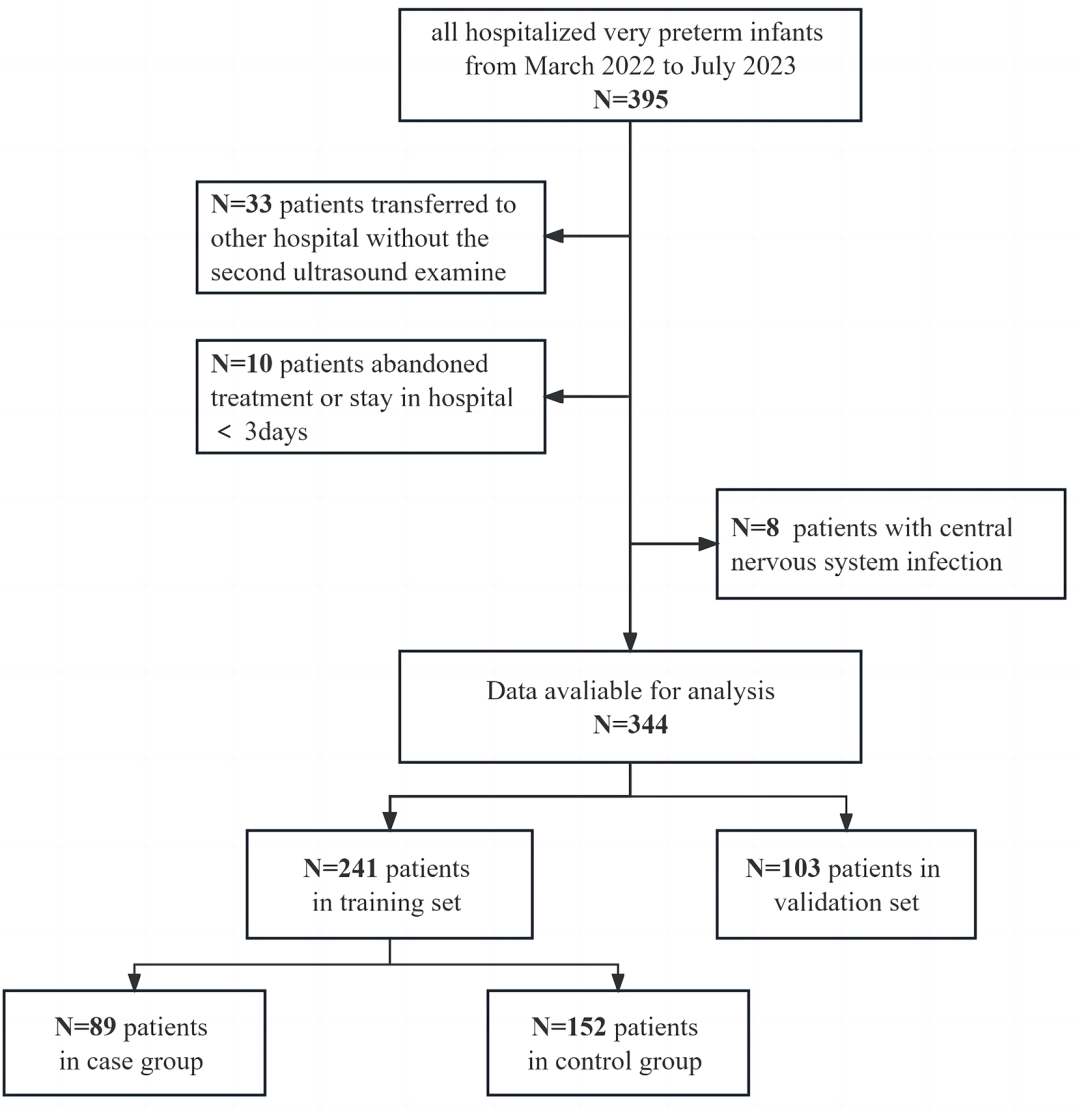
patients flow chart

We collected clinical records for all (*n* = 344) very preterm neonates hospitalized in Dongguan Maternal and Child Health Care Hospital from March 2022 to July 2023 (Fig. 1). This study received ethical approval from the hospital’s ethics committee, with the approval number 36 of 2022. We assigned 241 cases to the training set and 103 to the validation set (ratio 7:3). In training set, 89 patients with IVH, 152 patients were in control group.

#### Inclusion criteria

Neonates with a gestational age between 28 and 32 weeks were included in the study.

#### Exclusion criteria

Patients with the following comorbidities were excluded, congenital craniocerebral malformation, chromosomal disease, inherited metabolic disease, central nervous system infection, bilirubin encephalopathy, hypoglycemic encephalopathy. Additionally, patients with a hospital stay too short to allow for the second ultrasound examination were also excluded.

Transfontanelle ultrasonography times: The first transfontanelle ultrasonography was performed within 72 h of delivery, and a second ultrasound was performed 7–10 days after delivery.

Grade and type of ICH: Very preterm patients diagnosed with ICH were assigned to the case group. Patients free of ICH according to transfontanelle ultrasonography were assigned to the control group.

#### The types of ICH included

Periventricular intraventricular hemorrhage (PV-IVH): Classified into four grades according to Bowerman et al. [2]:

Grade I: Hemorrhage limited to the germinal matrix.

Grade II: IVH without ventricular dilatation.

Grade III: IVH with ventricular dilatation occupying > 50% of the ventricle.

Grade IV: IVH with intraparenchymal hemorrhage.

Grades III and IV are termed “severe ICH”.

Primary subarachnoid hemorrhage (SAH): Defined by the presence of blood in the subarachnoid space, observed on ultrasound.

Subdural hemorrhage (SDH): Identified by the presence of blood between the dura mater and the brain parenchyma.

Intraparenchymal hemorrhage (IPH): Defined by the presence of blood within the brain tissue itself.

Intracerebellar hemorrhage (ICH): Diagnosed based on the presence of blood within the cerebellum.

### Research methods

### Observation indicators

The following covariates were obtained from the medical records: sex, gestational age, birth weight, complications during gestation (gestational hypertension, diabetes mellitus, or anemia), mode of delivery (natural delivery, cesarean section), premature rupture of membranes (PROM), amniotic fluid, chorioamnionitis, birth asphyxia, number of embryos (single, twin, or multiple pregnancies), antenatal corticosteroids, maternal agnesium sulfate, administrationacidosis, intratracheal instillation of pulmonary surfactant (PS), and mechanical ventilation days, and .

#### To observe the probability of intracranial hemorrhage and the severity of this disease

#### To analyze the risk factors of intracranial hemorrhage in very preterm neonates

#### To analyze the single factors of intracranial hemorrhage in very preterm neonates

#### To develop a nomogram for ICH in very preterm neonates

### Statistical methods

We used SPSS (version 23.0) and R statistical software (version 4.3.2) for all data analysis. Normality of the distribution of numerical data was examined using Shapiro-Wilk test. Skewed numerical variables were presented as median (interquartile range), Inter group differences were compared using Mann Whitney-U test. Ordinal variables were expressed as frequencies and percentages (%), and comparisons between groups were performed using the Chi-squared test. Logistic regression analysis was used to analyze the independent risk factors. Prediction model made in R software, and the prediction accuracy and consistency of the model were assessed using the calibration curve, receiver operating characteristic curve (ROC), the area under the ROC curve (AUC), consistency index (C index), and decision curve. A p-value of less than 0.05 was considered statistically significant.

### Results

### General clinical characteristics

Between March 2022 and July 2023, 344 very preterm neonates were included, 241 in the training set and 103 in the validation set (Fig. 1). Of the 241 cases in the training dataset, 89 had intracranial hemorrhage (ICH) and 152 were controls. Clinical characteristics, including sex, gestational age, birth weight, and other relevant factors, are detailed in Table 1.

**Table 1:** Clinical characteristics and univariate comparison between the two groups

Characteristics	Case group(*n* = 89)	Control group(*n* = 152)	χ2/Zvalue	*P* value
Sex(male)	51(57.3)	91(59.9)	0.065	0.799
GA(weeks)	29.714(28.714, 30.643)	30.857(30.000, 31.429)	-6.159	**<0.001**
BW(g)	1240.0(970.0,1450.0)	1455.0(1230.0,1600.0)	-4.866	**<0.001**
Vaginal delivery	28(31.5)	56(36.8)	0.003	0.955
PROM(>18 h)	27(30.3)	48(31.6)	0.040	0.841
Amniotic fluid infection	14(15.7)	18(11.8)	0.438	0.508
Twin or tripple	13(14.6)	31(20.4)	0.902	0.342
Complications of gestation	16(18.0)	19(12.5)	0.951	0.329
Chorioamnionitis	3(3.4)	4(2.6)	0.109	0.742
Maternal agnesium sulfate administration	74(83.1)	138(89.5)	2.005	0.157
ACS	52(58.4)	112(73.7)	5.329	**0.021**
Birth asphyxia	15(16.9)	18(11.8)	0.807	0.369
Acidosis	47(52.8)	46(30.3)	11.108	**<0.001**
PS	72(80.9)	120(78.9)	0.039	0.843
Mechanical ventilation (days)	11.17(8.60, 16.89)	5.19(3.54, 11.16)	-4.723	**<0.001**

Data presented as number (%), median (interquartile range)

GA: gestational age; BW: birth weight; PROM: premature rupture of membranes; PS: intratracheal instillation of pulmonary surfactant; ACS: Antenatal corticosteroid

Mechanical ventilation defined as intubation-based ventilation in our study

Acidosis defined as arterial blood pH levels less than 7.35

Shapiro-Wilk test found that GA, BW and Mechanical ventilation days were not normally distributed, thus these covariates were analyzed using the Mann-Whitney U test, with results expressed as Z and p-values. The remaining risk factors were ordinal variables and analyzed using the Chi-square test, with results reported as χ2 and p-values

### Incidence and types of intracranial hemorrhage

There were 89 cases of intracranial hemorrhage in 241 very preterm neonates (36.9%), including 83 cases of PV-IVH (34.4%), among which 10 were grade III ~ IV. Additionally, there were 10 cases of severe PV-IVH, 5 cases of SAH, and one case of IPH (Table 2).

**Table 2:** Types and severity of intracranial hemorrhage

Types of brain injury	Case number	Probability(%)
Control group	152	63.1
PV-IVH	83	34.4
I ~ II grade	73	30.3
III ~ IV grade	10	4.1
SAH	5	2.1
IPH	1	0.4

PV-IVH: periventricular intraventricular hemorrhage; SAH: primary subarachnoid hemorrhage; SDH: subdural hemorrhage; IPH: intraparenchymal hemorrhage

### Univariate analysis of intracranial hemorrhage in very preterm neonates

There was no significant difference in sex, gestational complications, multiple pregnancies, mode of delivery, premature rupture of fetal membranes, amniotic fluid infection, birth asphyxia, or administration of pulmonary surfactant (every *P*>0.05) between cases with ICH compared to cases without. There were significant differences in GA (*P* < 0.001), BW (*P* < 0.001), ACS (*P* = 0.014), acidosis (*P* = 0.003), and mechanical ventilation days (*P* < 0.001).

**Fig. 2:**
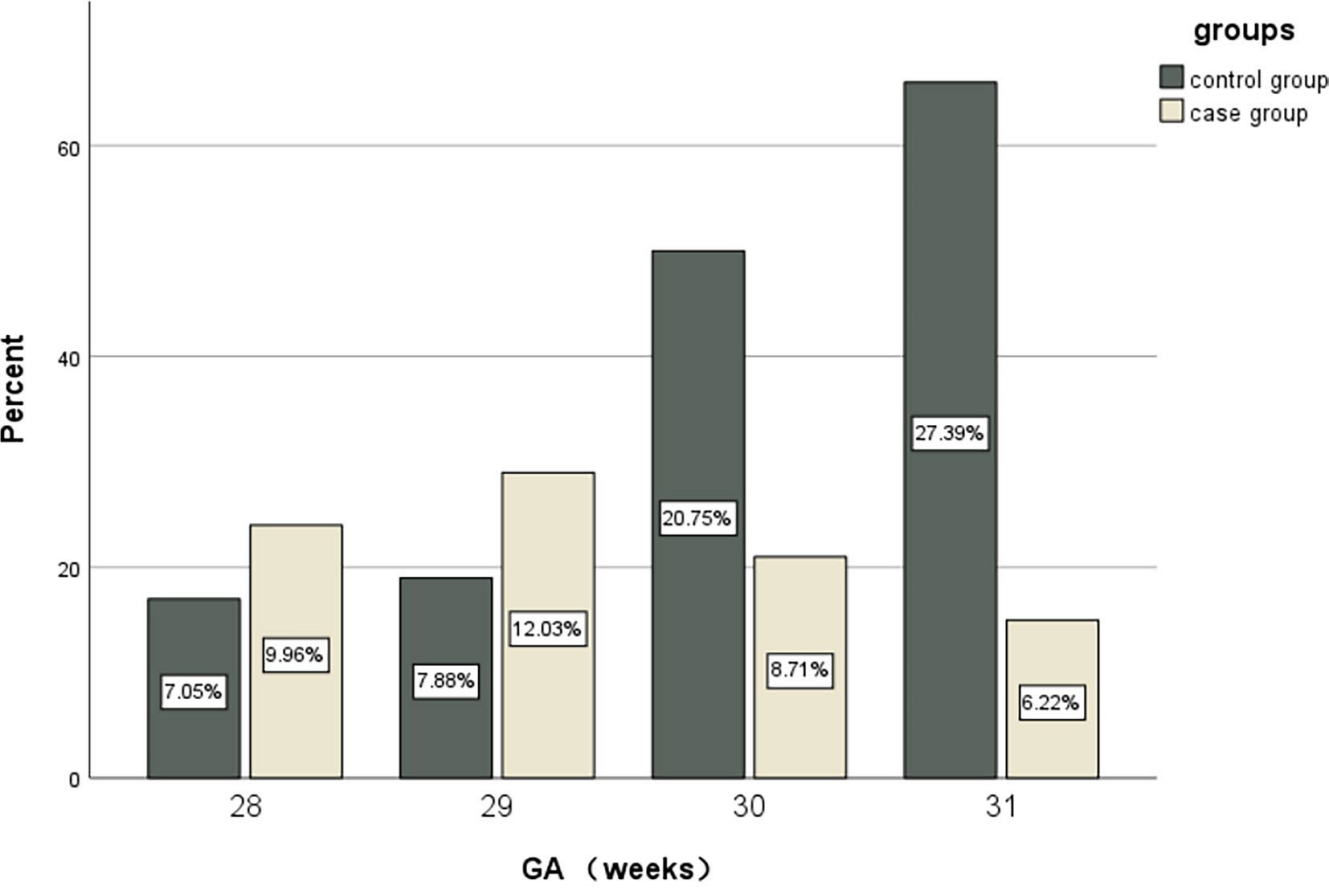
Percentage of neonates by gestational age in case and control groups

We also analyzed the data stratified by gestational age (Table 3). Intracranial hemorrhage occurred in 25.8% of the 41 neonates born at 28 weeks, 33.7% of the 48 neonates born at 29 weeks, 23.6% of the 71 neonates born at 30 weeks, and 16.9% of the 81 neonates born at 31 weeks. The incidence of ICH was significantly different among the four gestational age groups (χ2 = 33.022, *p*<0.001). Further pairwise comparisons between different GA groups revealed no significant difference between the 28 weeks group and the 29 weeks group. However, the 28 weeks group differed significantly from both the 30 and 31 weeks groups. Significant differences were also observed between the 29 weeks group and the 30/31 weeks groups. No significant difference was found between the 30 weeks and 31 weeks groups (Table 3; Fig. 2).

**Table 3:** Distribution of case and control groups by GA

GA(weeks)	Case group (*n* = 89)	Control group (*n* = 152)	χ2/P	Pairwise Comparisons χ2/P on GA(weeks)			
				28	29	30	31
28	24(27.0%)	17(11.2%)	33.022 *p*<0.001	/	0.032 *P* = 0.857	9.069 *P* = 0.003	20.045 *p*<0.001
29	29(32.6%)	19(12.5%)		/	/	11.180 *p*<0.001	23.541 *p*<0.001
30	21(23.6%)	50(32.9%)		/	/	/	2.560 *P* = 0.110
31	15(16.9%)	66(43.4%)		/			

GA: gestational age;

28 weeks include 28 ~ 28^+ 6^, 29 weeks include 29 ~ 29^+ 6^, 30 weeks include 30 ~ 30^+ 6^, 31 weeks include 31 ~ 31^+ 6^

We further conducted statistical analysis on the data based on the severity of acidosis (Table 4). We found a statistically significant difference between the intracranial hemorrhage (ICH) group and the control group across different levels of acidosis (*P* = 0.002). However, there was no statistically significant difference in the severity of periventricular-intraventricular hemorrhage (PVH-IVH) across different levels of acidosis (*P* = 0.791).

**Table 4:** Degree of acidosis between Control and Case groups

Degree of Acidosis	Control group(*n* = 152)	Case group(*n* = 89)			F/P	
		Mild PV-IVH(*n* = 73)	Severe PV-IVH(*n* = 10)	SAH + IPH(*n* = 6)	Case VS Control	Mild VS Severe PV-IVH
None	106(70.7%)	37(50.7%)	2(20.0%)	3(50.0%)	5.033 *P* = 0.002	0.348 *P* = 0.791
Mild	38(25.0%)	26(35.6%)	4(40.0%)	3(50.0%)		
Moderate	7(4.6%)	9(12.3%)	2(20.0%)	0(0%)		
Severe	1(0.7%)	1(1.4%)	2(20.0%)	0(0%)		

The severity of acidosis was graded by pH range: mild acidosis (7.30–7.35), moderate acidosis (7.20–7.29), and severe acidosis (less than 7.20)

### Multivariate analysis of intracranial hemorrhage in very preterm neonates

Logistic multivariate regression analysis identified acidosis (OR = 2.556, *P* = 0.004) and mechanical ventilation (OR = 0.257, *P* = 0.044) as independent risk factors for ICH in very preterm neonates, whereas ACS (OR = 0.248, *P* < 0.001) and increased GA (OR = 0.674, *P* = 0.020) were protective. Although there was significant difference in BW between case and controls in univariate analysis(Table 1), BW was not an independent risk factor for ICH in very preterm neonates(Table 5).

**Table 5:** Results of a multivariate logistic regression analysis

Items	β	OR	*P*	95%CI	
				Confidence lower limit	Confidence upper limit
GA	-0.394	0.674	**0.020**	0.484	0.939
ACS	-1.359	0.257	**<0.001**	0.129	0.512
Acidosis	0.938	2.556	**0.004**	1.360	4.803
Mechanical ventilation (days)	0.002	0.257	**0.044**	1.001	1.014
BW	-0.002	0.198	0.070	0.990	0.998

GA: gestational age; ACS: antenatal corticosteroid; BW: birth weight

### Intracranial hemorrhage prediction model

**Fig. 3:**
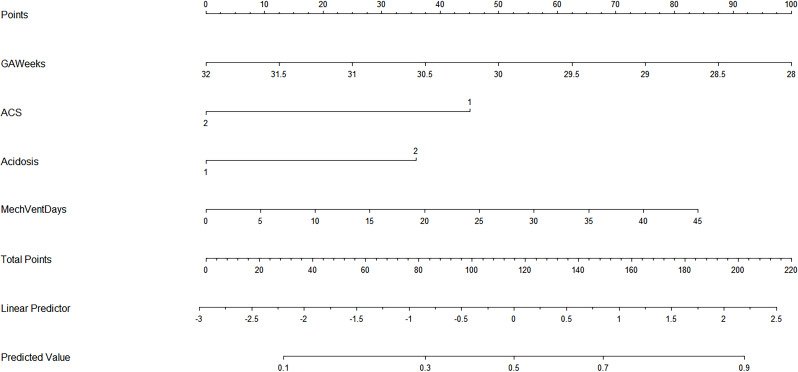
Nomogram for IVH prediction in very preterm neonates. GA: gestational age; ACS: antenatal corticosteroid; MechVent: mechanical ventilation; 1 stand for no and 2 stand for yes in ACS, Acidosis

In the multivariate logistic regression model, the following factors were associated with IVH: GA, ACS, Acidosis, and mechanical ventilation. We thus included these four variables in a prediction model and formed a nomogram to visualize the results of the regression analysis (Fig. 3).

**Fig. 4:**
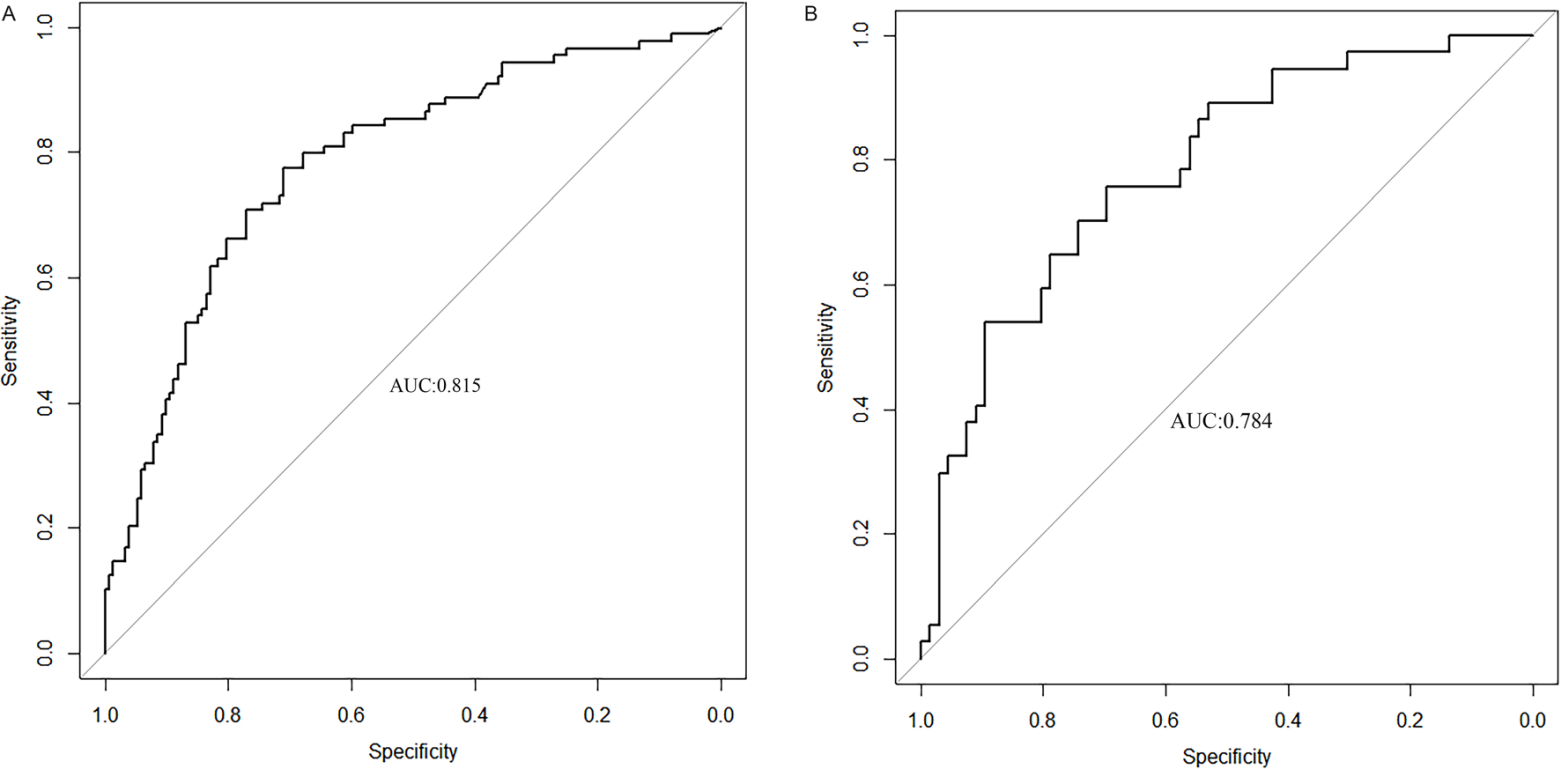
**a** ROC curve in training set Fig. 4b ROC curve in validation set. **B** ROC curves. ROC : receiver operating characteristic; AUC: area under the ROC curve

**Fig. 5:**
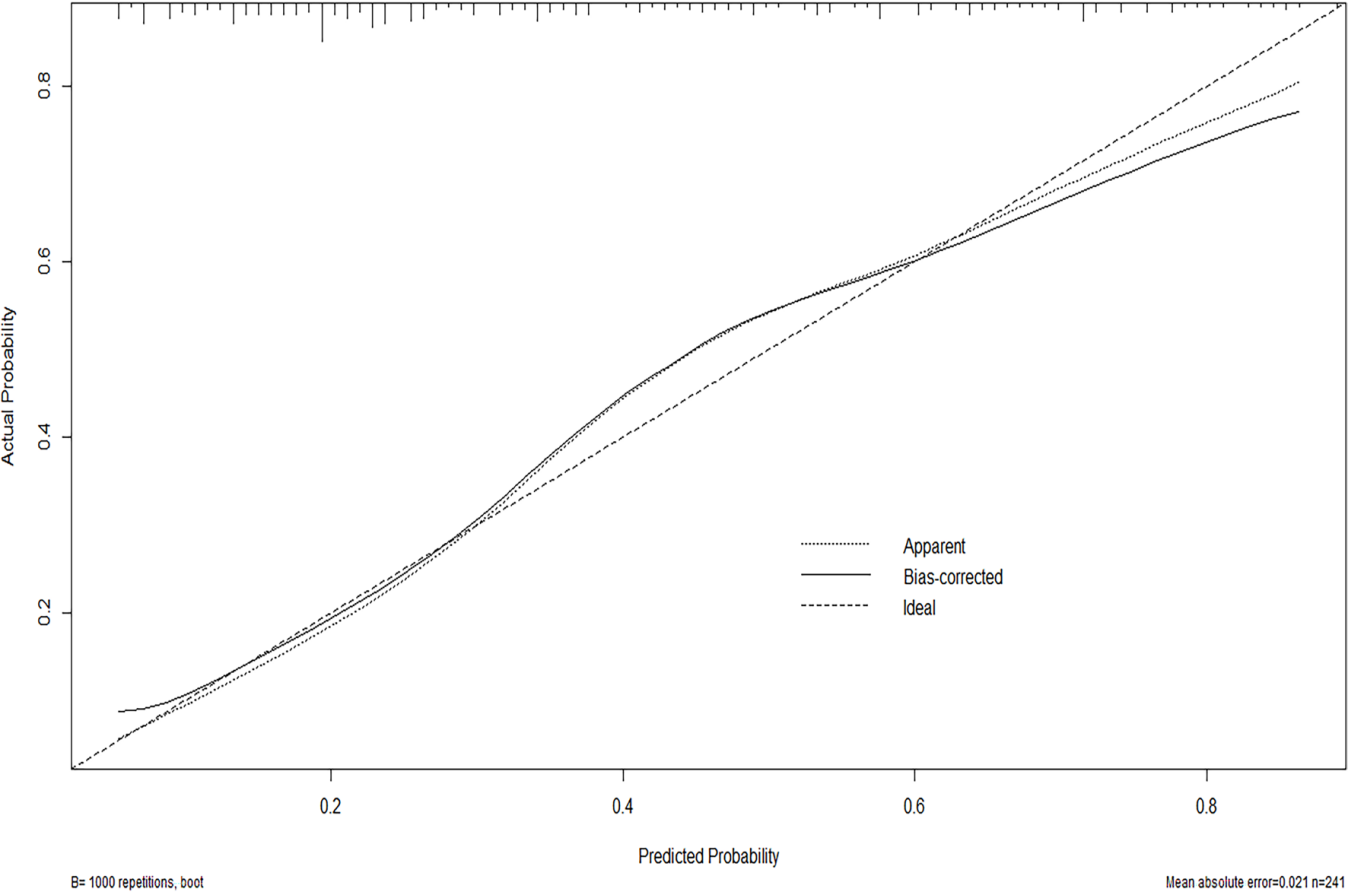
Calibration curve. In this curve, Mean absolute error = 0.021, Mean squared error = 0.00084, 0.9 Quantile of absolute error = 0.049

**Fig. 6:**
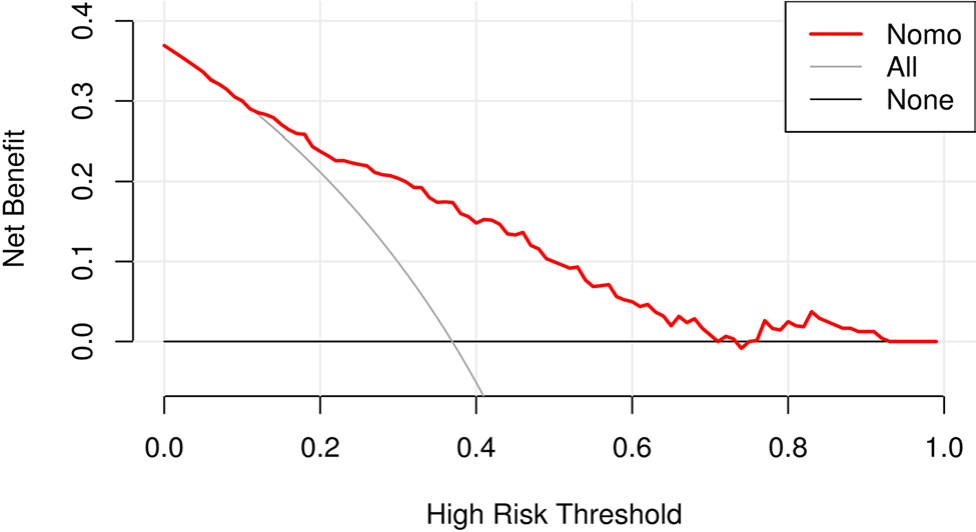
Decision curve. In this curve, our model is above the None and All lines within a relatively large range (0.12–0.92), indicating that the model has good clinical application value

We used ROC curve to assess the predictive accuracy of the nomogram. The C-index of the training and validation sets was 0.815 (95% CI:0.762–0.869) and 0.784 (95% CI: 0.693–0.875) respectively, indicating good discrimination (Fig. 4). The calibration curve of the predictive model used to assess the risk of ICH in preterms shows satisfactory consistency in this dataset (Fig. 5). Clinical application Decision analysis was performed on the data to assess the clinical usefulness of the prediction model. The analysis of the decision curve shows that the model can significantly improve clinical efficiency in predicting ICH (Fig. 6).

## Discussion

Intracranial hemorrhage commonly occurs in preterm neonates. It has been reported that the incidence of PV-IVH in very preterm, extremely preterm, very low and extremely low birth weight infants has been reported as high as 40%, with approximately 90% of cases occurring within the first 3 days of life [1, 3]. Reported risk factors for neonatal ICH include include sex, GA, mode of delivery, ACS, BW, hypoxia, hypercapnia, inflammation, application of PS, mechanical ventilation, etc [4, 5]. However, reported risk factors are not same across studies, even one exposure may behave as a risk factor or a protective factor in different studies. Previously, the population studied was mostly the entire preterm population or all newborns. Our study population is focused on the specific category of very preterm neonates, and we have expanded the risk factors to encompass 15 distinct variables. Based on this, we have established a nomogram model. A review of the research literature on neonatal brain injury shows that the probability of PV-IVH has gradually decreased in the all past 20 years around the world, from 50.9% in 1981 to 11.9% in Brazil, and about 10% in all premature infants in the United States in the past 10 years [6, 7]. But the ICH rate of patients in our study is not so low, because of the difference in the study population group. Therefore, our study more precisely reflects the incidence rate of very preterm neonates.

Our findings align with other studies, showing that the risk of ICH increases as GA decreases [1, 8, 9]. Preterm neonates are at risk for hemorrhage because of the abundance of stromal capillaries in the primary germinal matrix located under the ventricular canal in the lateral ventricles and a lack of connective tissue support. The thickness of germinal matrix is 2.5 mm at 23–24 weeks of gestational age, 1.4 mm at 32 weeks, and gradually faded by 36 weeks [10]. In addition, Preterm neonates have low blood perfusion in the terminal blood supply area and immature oligodendrocyte development, which can lead to damage of vascular endothelial cells, glial cells, axons and nerve myelin if combined with ischemia-reperfusion injury and oxygen free radical generation [4, 11, 12]. Our study compared the distribution of ICH cases across various gestational age weeks for very preterm neonates. In addition to identifying differences between several GA weeks, we found that the number of cases at 29 GA weeks was the highest, which has not been reported previously. This peak may be attributed to both a lower birth rate and a higher mortality rate among neonates born at GA earlier than 29 weeks. In this study, multivariate analysis did not find a correlation between BW and the occurrence of ICH in very preterm neonates, which is inconsistent with Zhu and others reports [13, 14]. Follow-up studies could be conducted in very low birth weight neonates or extremely low birth weight neonates to further determine the association between BW and ICH.

Our study showed long mechanical ventilation time was a high-risk factor for ICH, which was consistent with Cannavo’s conclusion [15]. Increased airflow can affect superior vena cava blood return, causing blood pressure fluctuations that can result in changes in intracranial pressure. In preterm infants, autoregulation of cerebral blood flow is not well developed, and direct impact of blood flow on the cerebrovascular wall can cause rupture and bleeding. It has been shown that altered cerebral blood flow occurs in neonates after mechanical ventilation, increasing the risk of brain injury [16, 17]. Our study only selected endotracheal intubation ventilatory support, and the commonly used neonatal continuous positive airway pressure respiratory support was not included, which may lead a limitation of the study. Different mechanical ventilation modes are used depending on the condition of the patient, making it difficult to categorize mechanical ventilation as a single covariate. The impact of various mechanical ventilation parameters on ICH in preterm infants deserves further study.

We found that acidosis was an independent risk factor for ICH in very preterm neonates, which was consistent with the findings of Ipsit’s and Meliss’s [18, 19]. Acidosis can impair a child’s cerebrovascular autoregulation, causing passive cerebral blood flow and potentially leading to cerebral hemorrhage in patients with high blood pressure. However, we observed that the severity of acidosis did not correlate with the incidence of ICH. This finding may be attributed to the prompt correction of acidosis in neonates at our hospital, which is typically achieved within a short timeframe.

ACS was a protective factor against ICH in preterm infants in our study, consistent with the findings of Elimian and others [20, 21]. Glucocorticoids exert anti-inflammatory effects to inhibit the inflammatory cascade response, and glucocorticoids promote the developmental maturation of oligodendrocytes and endothelial cells to exert neuroprotective effects. In experimental animal studies, glucocorticoids promote the maturation of capillaries in the murine choroid plexus [22]. One study reported that the timing of the last dose of corticosteroid before delivery also influences risk for brain injury, with significantly reduced risk observed when the interval since the last dose is greater than 48 h, compared with less than 24 h [23]. On the contrary, prenatal dexamethasone also can damage neural cells in the newborn and even have a negative effect on the distant neurodevelopment of the children [24]. We need to think about the pros and cons and evaluate the timing of the use of dexamethasone in the prevention of ICH in very preterm infants.

Few studies have developed nomograms for ICH in very preterm neonates. Our model, through internal validation, shows good identification and calibration capabilities, which can aid in the early prevention and intervention of ICH. However, in Clinical Application Decision Analysis, the model is found to perform better within the range of moderate threshold probabilities, but its accuracy diminishes at high or very low probabilities. To refine this model, it’s necessary to incorporate additional factors such as coagulation functions, sepsis, etc [25, 26].

Our study had some limitations. First, our research was conducted solely at our hospital, if data from more research centers were included, our results would more accurately reflect the ICH conditions of very preterm neonates. Second, our study population was limited to gestational ages between 28 and 32 weeks, excluding extremely preterm neonates and those with even younger gestational ages, which may reduce the clinical applicability of our study. As our center’s capacity for treating extremely preterm infants at 27 weeks and below gradually improves, we plan to include this population in our future research.

In conclusion, we investigated the relationship between very preterm ICH and predictors: Our nomogram showed good accuracy in assessment of ICH risk in preterms. This model can be used to select infants at risk for ICH and make clinical decisions like performing cranial ultrasound in these for confirmation while avoiding excessive interventions for low-risk neonates. However, external verification and more factors are still required in the future.

## Electronic supplementary material

Below is the link to the electronic supplementary material.

Supplementary Material 1

Supplementary Material 2

## Data Availability

Data is provided within the manuscript or supplementary information files.
